# Supplementary material for: Mycn Is Essential for Pubertal Mammary Gland Development and Promotes the Activation of Bcl11b-Maintained Quiescent Stem Cells
Source: Cells. 2025 Aug 12;14(16):1239. doi: 10.3390/cells14161239 (PMC12384323; doi:10.3390/cells14161239)
Supplement: Supplementary file 1 [file cells-14-01239-s001.zip › cells-3771693-supplementary.pdf]

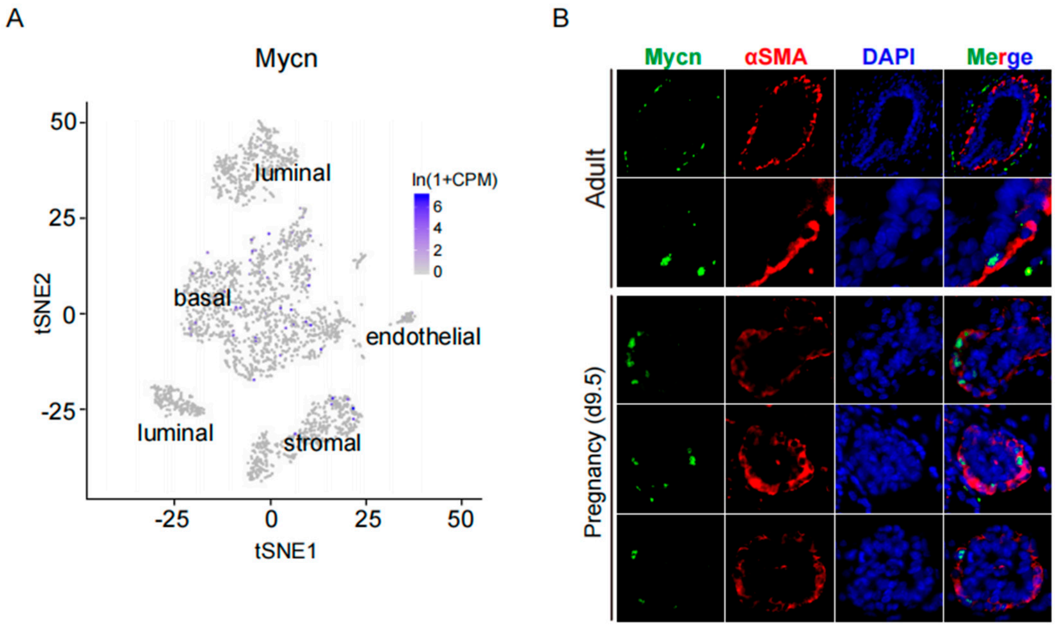

Supplementary Figure S1. The expression of Mycn in adult (duct) and pregnant (alveolar) stage in mouse mammary. (A) t-SNE plot shows the expression of *Mycn* in adult mouse mammary (data from Tabula Muris database, https://tabulamuris.sf.czbiohub.org/visualizations). (B) Representative immunofluorescence imaging of the expression of Mycn in TEB and adult duct and alveolar basal cells. Magenta/GFP: Mycn; Red: αSMA; blue: DAPI.

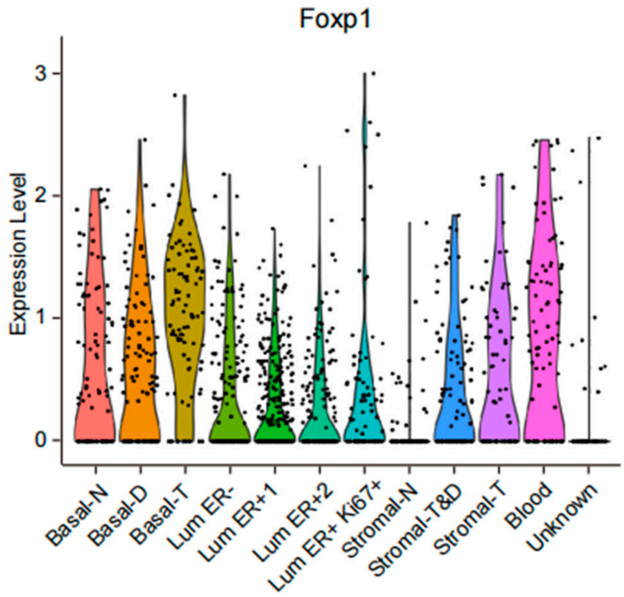

Supplementary Figure S2. The expression of Foxp1 in pubertal mouse mammary. Violin plots show the single-cell expression levels of Foxp1 in pubertal mouse mammary tissue. Foxp1 is highly expressed in most basal cells and a significant number of luminal cells.

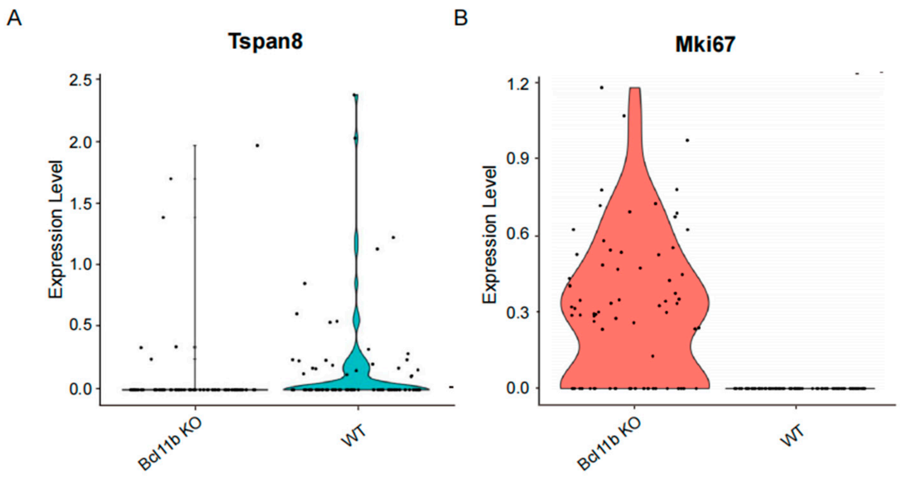

Supplementary Figure S3. *Bcl11b* KO decreased the expression level of Tspan8 and activated cell proliferation. (A-B) Violin plots show the single-cell expression level of Tspan8 (A) and Mki67 (B) in 4-month-old adult control and *Bcl11b* KO mice under C57BL/6 background.

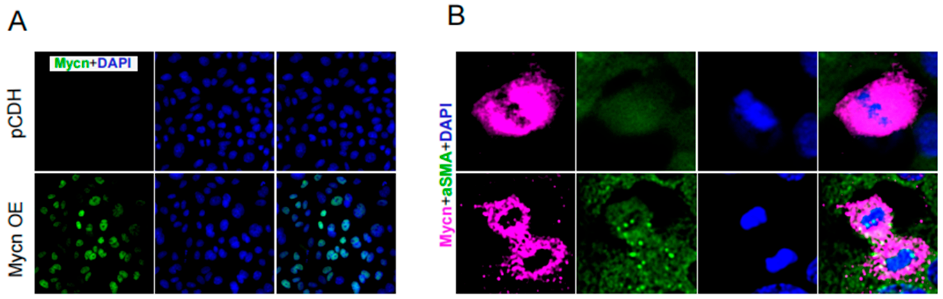

Supplementary Figure S4. Detect the expression of Mycn in cultured mammary basal cells. (A) Representative immunofluorescence images showing the expression of Mycn in *Mycn*-overexpressed primary basal cells from pubertal mouse mammary. GFP: Mycn; blue: DAPI. (B) Representative immunofluorescence images showing Mycn's potential involvement in spindle behavior during primary basal cell division. Magenta: Mycn; purple: αSMA; blue: DAPI.

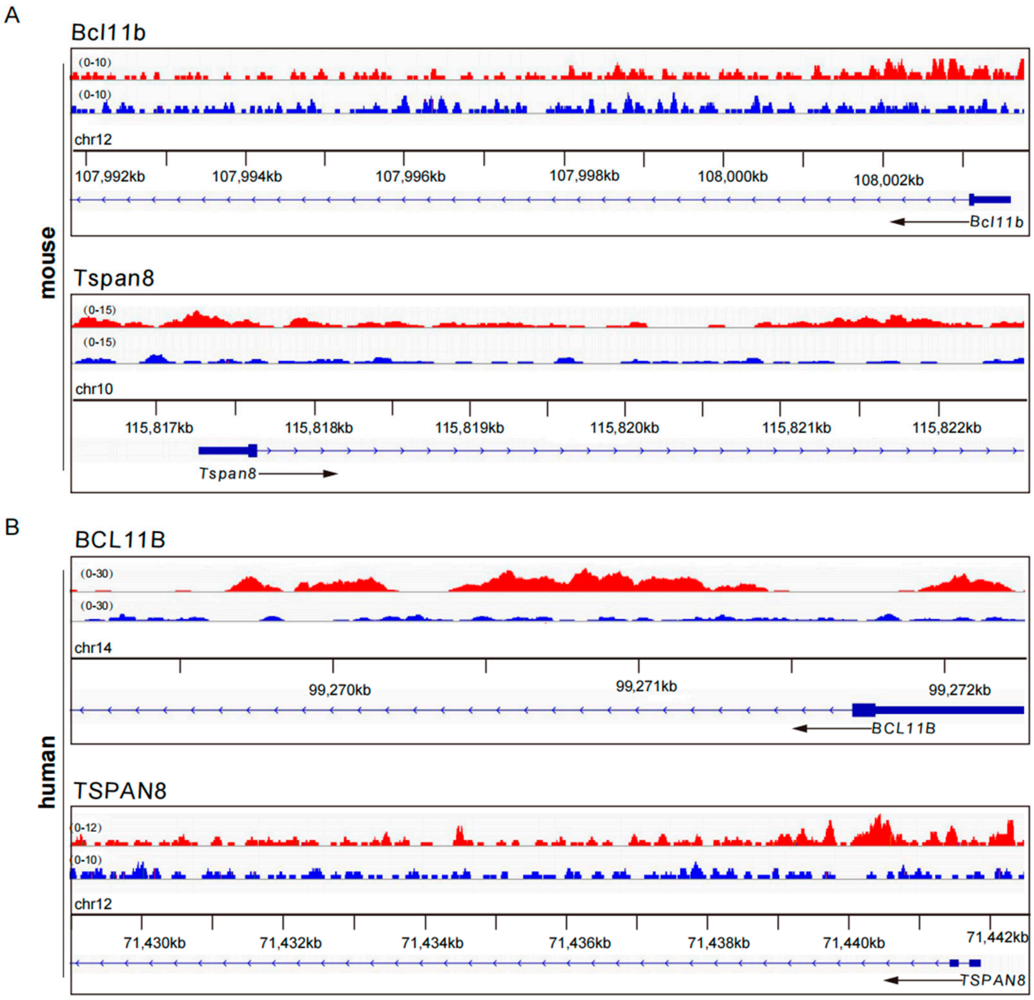

Supplementary Figure S5. ChIP-Seq data showing Mycn's (MYCN in human) direct interaction with Bcl11b and Tspan8 (BCL11B and TSPAN8 in human) in both mouse and human neuroblastoma. (A) ChIP-Seq data for Mycn's direct interaction with Bcl11b and Tspan8 in mouse neuroblastoma. The data originates from the published GEO database under the accession number GSE151426. (B) ChIP-Seq data for MYCN's direct interaction with BCL11B and TSPAN8 in human neuroblastoma. The data originates from the published GEO database under the accession number GSE94782.
